# Volume Status Assessment by Lung Ultrasound in End-Stage Kidney Disease: A Systematic Review

**DOI:** 10.1177/20543581231217853

**Published:** 2023-12-25

**Authors:** Aileen Kharat, Faissal Tallaa, Marc-Antoine Lepage, Emilie Trinh, Rita S. Suri, Thomas A. Mavrakanas

**Affiliations:** 1 Division of Respirology, Geneva University Hospitals, Switzerland; 2 Division of Nephrology, Department of Medicine, McMaster University, Hamilton, ON, Canada; 3 Division of General Internal Medicine, Department of Medicine, McGill University Health Centre, Montreal, QC, Canada; 4 Division of Nephrology, Department of Medicine, McGill University Health Centre, Montreal, QC, Canada

**Keywords:** lung ultrasound, chronic kidney disease, volume assessment, dialysis, mortality, major adverse cardiovascular events

## Abstract

**Purpose of review:**

Lung ultrasound is a noninvasive bedside technique that can accurately assess pulmonary congestion by evaluating extravascular lung water. This technique is expanding and is easily available. Our primary outcome was to compare the efficacy of volume status assessment by lung ultrasound with clinical evaluation, echocardiography, bioimpedance, or biomarkers. The secondary outcomes were all-cause mortality and cardiovascular events.

**Sources of information:**

We conducted a MEDLINE literature search for observational and randomized studies with lung ultrasound in patients on maintenance dialysis.

**Methods:**

From a total of 2363 articles, we included 28 studies (25 observational and 3 randomized). The correlation coefficients were pooled for each variable of interest using the generic inverse variance method with a random effects model. Among the clinical parameters, New York Heart Association Functional Classification of Heart Failure status and lung auscultation showed the highest correlation with the number of B-lines on ultrasound, with a pooled *r* correlation coefficient of .57 and .36, respectively. Among echocardiographic parameters, left ventricular ejection fraction and inferior vena cava index had the strongest correlation with the number of B-lines, with a pooled *r* coefficient of .35 and .31, respectively. Three randomized studies compared a lung ultrasound-guided approach with standard of care on hard clinical endpoints. Although patients in the lung ultrasound group achieved better decongestion and blood pressure control, there was no difference between the 2 management strategies with respect to death from any cause or major adverse cardiovascular events.

**Key findings:**

Lung ultrasound may be considered for the identification of patients with subclinical volume overload. Trials did not show differences in clinically important outcomes. The number of studies was small and many were of suboptimal quality.

**Limitations:**

The included studies were heterogeneous and of relatively limited quality.

## Abrégé

**Motif de la revue:**

L’échographie pulmonaire est une technique non-invasive réalisée au chevet du patient qui permet d’évaluer avec précision la congestion pulmonaire en mesurant l’eau pulmonaire extravasculaire. Cette technique facilement accessible est de plus en plus utilisée. Notre principal critère de jugement était de comparer l’efficacité de l’évaluation de la volémie par échographie pulmonaire avec l’évaluation clinique, l’échocardiographie, la bio-impédance ou les biomarqueurs. Les critères d’évaluation secondaires étaient la mortalité toutes causes confondues et les événements cardiovasculaires.

**Sources:**

Nous avons recherché sur MEDLINE les études observationnelles et les essais randomisés où une échographie pulmonaire avait été réalisée chez des patients sous dialyse d’entretien.

**Méthodologie:**

Sur un total de 2 363 articles, nous avons retenu 28 études (25 observationnelles et 3 randomisées). Les coefficients de corrélation ont été regroupés pour chaque variable d’intérêt en utilisant la méthode générique de variance inverse avec un modèle à effets aléatoires. Les paramètres cliniques qui avaient montré les corrélations les plus élevées avec le nombre de lignes B à l’échographie étaient le statut de l’insuffisance cardiaque selon la classification de la New York Heart Association et l’auscultation pulmonaire, avec des coefficients de corrélation r regroupés respectifs de 0,57 et de 0,36. Les paramètres de l’échocardiographie qui avaient montré les plus fortes corrélations avec le nombre de lignes B étaient la fraction d’éjection du ventricule gauche et l’indice de la veine cave inférieure, avec des coefficients r regroupés respectifs de 0,35 et de 0,31. Trois essais randomisés avaient comparé une approche guidée par échographie pulmonaire aux normes de soins selon des critères cliniques stricts. Bien que les patients du groupe avec échographie pulmonaire aient montré une décongestion plus efficace et un meilleur contrôle de la pression artérielle, aucune différence n’a été observée entre les deux stratégies de prise en charge en ce qui concerne les décès de toutes causes confondues ou les événements cardiovasculaires indésirables majeurs.

**Principales observations:**

L’échographie pulmonaire pourrait être envisagée pour identifier les patients qui présentent une surcharge volumique subclinique. Les essais inclus n’ont pas montré de différences dans les résultats cliniquement pertinents. Le nombre d’études incluses était faible et plusieurs étaient de qualité sous-optimale.

**Limites:**

Les études incluses étaient hétérogènes et de qualité relativement limitée.

## Introduction

Chronic kidney disease (CKD) is a common condition and is often associated with pulmonary congestion.^1,2^ Pulmonary congestion in advanced CKD is associated with higher cardiovascular morbidity and mortality, compared with patients with CKD and optimal volume status.^3,4^ Therefore, volume status management is an important part of the standard of care in these patients.

Assessment of volume status can be challenging, and clinical assessment is often imprecise.^5,6^ Therefore, different adjunctive diagnostic tools are used in clinical practice, such as bioelectrical impedance, chest radiography, weight monitoring, and blood biomarkers.^7-10^ More recently, lung ultrasound has been proposed for the assessment of extravascular lung water and therefore reflects lung congestion.^11^

Since the 1990s, there has been growing interest in lung ultrasound. First, it was used for critically ill patients in intensive care units and it has now expanded to most fields in modern medicine. This technique is simple, reproducible, radiation free, and can be easily performed at the bedside.^12,13^ The presence and number of B-lines artifacts is considered a surrogate for alveolar interstitial syndrome, as first described by Lichtenstein et al.^14^ It is now a validated tool for the estimation of volume overload in patients with heart failure.^15,16^ The presence of a B-line pattern and pleural effusions visualized by lung ultrasound is highly suggestive of volume overload.

In the nephrology literature, many articles have been published on the prognostic value of lung ultrasound in patients undergoing hemodialysis.^17,18^ The recent publication of the LUST study provided interesting data on the added value of lung ultrasound in patients with CKD.^19^ An excellent meta-analysis published in 2019 reported the technological adjuncts for volume status management and the effect on mortality. The primary outcome presented was mortality and numerous tools were assessed. However, there was no comparison between the various techniques.^20^ Therefore, we conducted this systematic review including the most recent data to present current evidence on use of lung ultrasound in this setting. We are comparing the efficacy of lung ultrasound to clinical evaluation and other standard techniques, such as cardiac ultrasound, blood biomarkers, and bioimpedance, commonly used to assess fluid status in this group of patients. We will also assess the impact of timely diagnosis of volume overload by lung ultrasound on cardiovascular events and mortality.

## Materials and Methods

### Search Strategy

We conducted a MEDLINE literature research in PubMed for relevant literature through January 2023. We searched for published clinical trials in English or French language, including patients of at least 18 years of age. The key words used for literature research in PubMed were (pulmonary ultrasound) OR (lung ultrasound)) AND ((dialysis) OR (end-stage kidney disease)) OR (kidney failure) OR (chronic kidney disease)).

Available meta-analyses were also reviewed. We verified the reference list of retrieved articles and had notifications set from PubMed for new publications. The protocol was registered in the PROSPERO registry in July 2020 (CRD42020197765). The results are reported using the Preferred Reporting Items for Systematic Reviews and Meta-Analyses (PRISMA) checklist (Supplementary File 1).

### Eligibility Criteria

All the following criteria should apply: (1) study population: adult patients with advanced CKD defined as an estimated glomerular filtration rate (eGFR) < 30 mL/min/1.73 m^2^, including patients with end-stage kidney disease (ESKD) undergoing maintenance dialysis (hemodialysis and peritoneal dialysis); (2) intervention: use of lung ultrasound to assess volume status and/or guide volume management; (3) study design: randomized controlled trials (RCTs) or observational (cohort) studies, published in the form of an article or abstract; (4) at least, one of the relevant outcomes should be reported: (a) mortality, (b) correlation with volume status assessment by other methods, such as clinical evaluation, bioimpedance, biomarkers, or echocardiography, and (c) admission for heart failure—volume overload.

### Study Selection and Quality Assessment

Two authors (AK and FT) independently performed study selection and extracted relevant information from the included trials. Discrepancies between author assessments were resolved by mutual discussion of each item in question. In case of disagreement, this was discussed in a conference with the senior author (TM). To assess the quality of included studies, the second version of the Cochrane risk-of-bias tool for RCTs (RoB2) was used for randomized control trials, the Newcastle-Ottawa scale for cross-sectional studies, and the Robins-I tool for all other observational studies (shown in Supplementary Files 2 and 3).^21-23^

### Outcomes

Our primary outcome was to compare the efficacy of volume status assessment by lung ultrasound with clinical evaluation, echocardiography, and paraclinical parameters (bioimpedance and biomarkers).

For echocardiography, left ventricular ejection fraction (LVEF), left ventricular mass index (LVMI), the E/é ratio measured by tissue Doppler imaging, the inferior vena cava (IVC) index, and the right ventricular systolic pressure were used. These parameters are validated measures to estimate volume status.^15^ To reduce heterogeneity and better reflect clinical significance, only echocardiography examinations performed before the beginning of the dialysis session were used.

The secondary outcomes were all-cause or cardiac mortality and heart failure admissions with lung ultrasound-guided or standard techniques.

### Statistical Analysis

Results are reported according to the PRISMA 2009 checklist (Supplementary File 1).^24^ The correlation coefficients were pooled for each variable of interest after having been transformed to *z* values.^25^ When both the Pearson and Spearman coefficients were available, Pearson coefficients were used. When both the predialysis and postdialysis session coefficients were available, the predialysis (prior to the beginning of the dialysis session) coefficients were used. Pooled *z* values were calculated using the generic inverse variance method with a random effects model. The *I*^2^ index was used to quantify heterogeneity and assess inconsistency. Pooled *z* values and 95% confidence intervals were then back transformed to *r* values and respective 95% confidence intervals. For the null hypothesis of no correlation, the *t* distribution was used with n − 2 degrees of freedom.^25^ Statistical analyses were performed in Stata (version 14 IC; College Station, Texas).

## Results

### Study Selection

**Figure 1.**
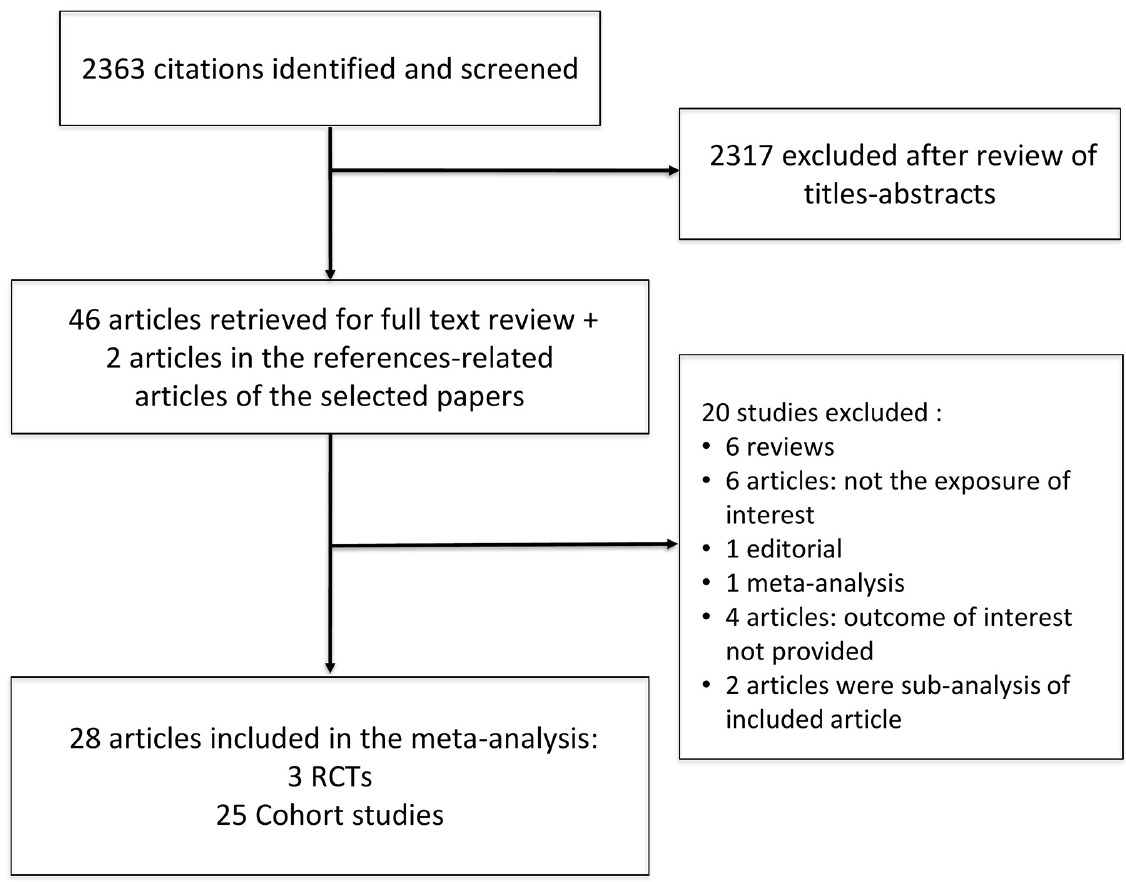
Study flowchart. *Note.* RCT = randomized controlled trial.

We retrieved 2363 articles on lung ultrasound in CKD in our primary search. A total of 2317 articles were excluded after title and abstract review. We also identified 2 articles in the reference list of manuscripts selected for full-text review, while 2 articles were retrieved from publication alert e-mails from PubMed received after our initial search. From the 46 articles selected for full-text review, we excluded another 20 articles: 5 review articles (all studies mentioned in these manuscripts had already been included in our review), 1 editorial, 1 meta-analysis, 6 articles that did not study the exposure of interest, 4 articles that did not present the outcome(s) of interest, 2 articles were subanalyses of an included randomized controlled study. A total of 28 studies were included in our systematic review: 25 observational studies,^4,26-49^ and 3 RCTs as shown in Figure 1.^18,19,50^ The randomized controlled studies did not address the same endpoints as the observational studies and are separately presented in a different section of the results. A meta-analysis of randomized controlled studies was not performed because of the small number of trials, the different endpoints, and the distinct populations enrolled.

Even though the study by Torino et al^41^ is an RCT, only the results of the intervention arm are reported in our analysis, and this is why it was considered an observational study in this review. All studies enrolled patients on maintenance dialysis (5 of them enrolled patients on peritoneal dialysis). There were no relevant studies in patients with advanced renal disease not on maintenance dialysis.^35,37^ Baseline characteristics of the included studies are shown in Table 1.

**Table 1.** Study Characteristics.

Article number of patients, n	Study design	Country	Age	Male sex, %	Modality	Co-morbidities				LUS method	Comparison arm	Quality assessment
						CHF	CAD	HTN	DM2			
Annamalai et al^26^ (n = 50)	Cross-sectional study	India	37 ± 11	74	HD	N/A	N/A	N/A	14%	28 points Sum of B-lines (B-lines score or comet score)	Edema Dyspnea	3/6 NewOtt
Arun Thomas et al^27^ (n = 79)	Prospective cohort study	India single center	54 ± 12	81	HD	N/A	N/A	N/A	45%	8 zones Sum of B-lines	IVC	See supplement
Basso et al^28^ (n = 30)	Cross-sectional Study	Italy	64 ± 16	70	HD	N/A	74%	77%	16%	28 points Sum B-lines	BIS IVC	3/5 NewOtt
Beaubien-Souligny et al^29^ (n = 47)	Prospective cohort study	Canada Unicentric MTL	67 ± 14	64	HD	N/A	N/A	91%	53%	28 regions B-lines YES/NO 0/28 points	Dry weight assessment	See supplement
Bobot et al^43^ (n = 31)	Prospective study	France	63 ± 13	71	HD	19%	41%	84%	35%	28 points Sum B-lines	Clinical score (NYHA, orthopnea, crackles, peripheral edema, jugular turgor, hepatic-jugular reflex, pre-HD BP) TTE (IVC, sPAP, E/é, E/A)	See supplement
Donadio et al^30^ (n = 31)	Prospective study	Pisa Italy	68 ± 11	72	HD	N/A	32%	87%	45%	57 points Sum of B-lines	BIA total and thoracic (TBW, ECW, ECWI, TBWI)	See supplement
Fornazarič et al^44^ (n = 16)	Prospective observational study	Slovenia	53 ± 18	56	PD	N/A	N/A	N/A	N/A	28 points Sum of B-lines	IVC BIS NT-proBNP	See supplement
Giannese et al^45^ (n = 24)	Prospective cohort study	Italy	60 ± 18	63	HD	N/A	N/A	71%	25%	28 points Sum of B-lines	BIA (TBW, ECW, ICW, AFO, RFO) BNP	See supplement
Jiang et al^31^ (n = 20)	Prospective study	NY, USA	66 ± 14	55	HD	N/A	N/A	35%	35%	28 points Sum of B-lines	Dry weight	See supplement
Kawachi et al^32^ (n = 61)	Prospective observational study	Japan	75 ± 11	52	HD	N/A	N/A	87%	46%	8 areas	IVC TTE (LVEF)	See supplement
Loutradis et al^51^ (n = 71)	RCT (Sub-LUST)	Greece and Slovenia	63 ± 14	66	HD	24%	38%	100%	27%	28 points Total B-lines from 0 to 280	BP	See supplement
Mallamaci et al^33^ (n = 75)	Cross-sectional	Italy	63 ± 23	65	HD	N/A	43%	56%	N/A	Lung comet and Lung comet score 28 points	NYHA TTE (E/é, LVEF, PAPs, LVMI)	3/5 NewOtt
Mohammad et al^47^ (n = 38)	Cross-sectional	Saudi Arabia	46 ± 14	88	HD	N/A	N/A	47%	21%	28 points Total B-lines	Clinical score (BP, RR, orthopnea, JVP, crackles, peripheral edema, ascites, pleural effusion, NYHA) IVC	3/5 NewOtt
Ngoh et al^34^ (n = 50)	Cross-sectional	Singapore	59 ±14	47	HD	N/A	30%	85%	73%	28 points Total B-lines	BIS (TBW, ECW, ICW, ΔHS)	4/6 NewOtt
Pannucio et al^35^ (n = 88)	Cross-sectional study	Italy Multicenter	61 ± 17	50	PD	N/A	41%	N/A	23%	Lung comet score	BIA TTE	4/5 NewOtt
Pardała et al^36^ (n = 54)	Cross-sectional study	Poland	58	59	HD	N/A	57%	93%	28%	28 points B-lines 0-10 per scanning zone Sum of those B-lines	BIA (RFO) TTE (RVSP/LVEF, LVMI, RAVI/LAVI) IVC min/max	3/5 NewOtt
Paudel et al^37^ (n = 27)	Cross sectional	UK	62 ± 3	63	PD	N/A	33%	N/A	33%	28 points Sum of B-lines (or ULC)	BIS	3/4 NewOtt
Saad et al^38^ (n = 81)	Cross-sectional study	Staten Island USA	60 ± 16	72	HD	N/A	N/A	94%	52%	28 points Comet tail score (=Sum B-lines)	TTE (E/é, LVEF)	6/6 NewOtt
Santos et al^39^ (n = 73)	Cross-sectional	Brazil (2 centers)	61 ± 16	63	HD	N/A	N/A	N/A	100%	28 points B-lines number	BIA (ECW, FO, RFO [FO/ECW x100])	5/5 NewOtt
Sevinc et al^48^ (n = 21)	Cross-sectional	Turkey	48 ± 10	19	PD	N/A	19%	10%	10%	28 points Sum B-lines	Clinical (orthopnea, PND, NYHA, crackles, S3, peripheral edema) CXR TTE (LVEDV, LVMI, LVEF, sPAP, E/é) BIA	3/5 NewOtt
Siriopol et al^40^ (n = 96)	Prospective observational Study	Romania	59 ± 14	51	HD	N/A	N/A	N/A	24%	Lung comet count 28 points	BIS (TBW, ECW, ICW, ΔHS)	See supplement
Siriopol et al^18^ (n = 250)	RCT	Romania	59 ± 14	46	HD	N/A	16%	76%	19%	28 points Sum B-lines (called BLS for B-lines score)	BIS (AFO, RFO, TBW, ECW, ICW)	See supplement
Lučič Šrajer et al^46^ (n = 19)	Cross-sectional	Slovenia	54 ± 24	63	PD	N/A	N/A	N/A	N/A	28 points Sum B-lines	Crackles Peripheral edema NT-proBNP	4/5 NewOtt
Torino et al^41^ (n = 79)	Prospective study	Italy	72	65	HD	N/A	100%	N/A	37%	28 points 4 groups		See supplement
Trirattanapikul et al^49^ (n = 20)	Prospective cohort study	Thailand	62 ± 14	70	HD	N/A	N/A	N/A	45%	28 points Sum B-lines	BIS (TBW, ECW, ICW, AFO, RFO) Dry weight	See supplement
Weitzel et al^42^ (n = 20)	Cross-sectional	USA Michigan	53 ± 14	85	HD	5%	20%	95%	55%	Comet count	—	3/5 NewOtt
Zoccali et al^4^ (n = 392)	Cohort study multicenter	Italy	65 ± 15	63	HD	N/A	55%	56%	29%	28 points	—	See supplement
Zoccali et al^19^ (n = 363)	RCT	Multicentric	70 ± 11	70	HD	43%	72%	76%	41%	Lung comet score	TTE (LVMI, LVEF, E/é)	See supplement

*Note.* Age is reported as mean ± standard deviation. LUS = lung ultrasound; CHF = congestive heart failure; CAD = coronary artery disease; HTN = hypertension; DM2 = diabetes mellitus type 2; HD = hemodialysis; N/A = not available; NewOtt = Newcastle and Ottawa score; IVC = inferior vena cava; BIS = bioimpedance spectroscopy; NYHA = New York Heart Association; BP = blood pressure; TTE = transthoracic echocardiography; PAPs = pulmonary arterial pressures; E/é = early filling to early diastolic mitral annular velocity; BIA = bioelectrical impedance analysis; TBW = total body water; ECW = extracellular water; ECWI = extracellular water index; RCT = randomized-controlled trial; TBWI = total body water index; PD = peritoneal dialysis; ICW = intracellular water; AFO = absolute fluid overload; RFO = relative fluid overload; BNP = brain natriuretic peptide; LVEF = left ventricular ejection fraction; LVMI = left ventricular mass index; ΔHS = hydration status; RVS = right ventricular systolic pressure; RAVI = right atrial volume indexed for BSA; LAVI = left atrial volume indexed for BSA; ULC = ultrasound lung comets; FO = fluid overload; LVEDV = left ventricular end diastolic volume; MTL = Montreal; RR = Respiratory rate; JVP = Jugular venous pressure; PND = Paroxystic nocturnal dyspnea; CXR = Chest xray.

### Correlation Between Lung Ultrasound and Clinical Parameters

**Figure 2.**
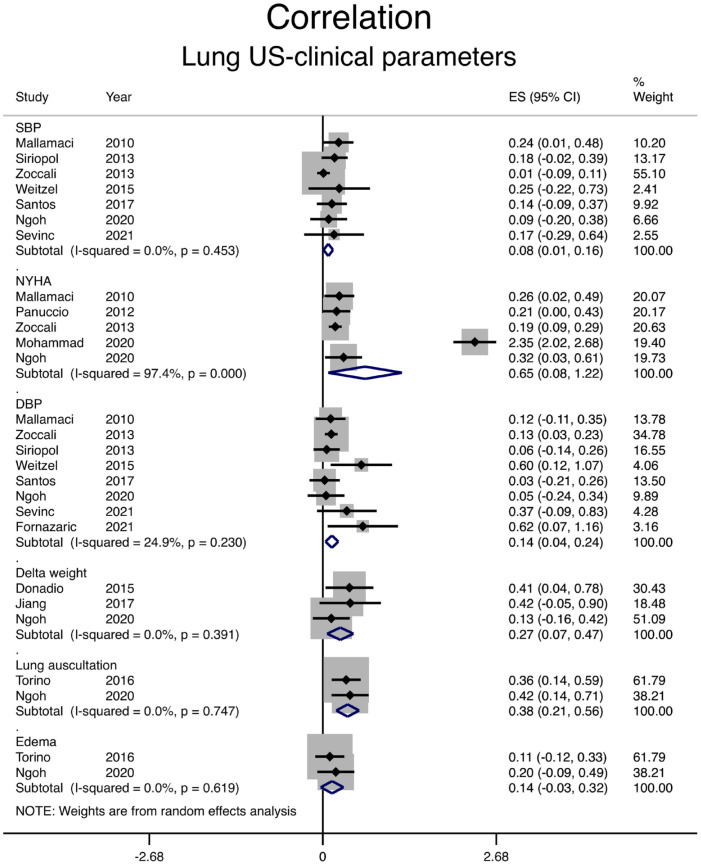
Correlation between the number of B-lines on lung ultrasound and clinical parameters. *Note.* SBP = systolic blood pressure; CI = confidence interval; NYHA = New York Heart Association; DBP = diastolic blood pressure; ES = Effect size.

Correlation between the number of B-lines on lung ultrasound and different clinical parameters is shown in Figure 2 and Table 2. Systolic and diastolic blood pressure (BP) and peripheral edema poorly correlate with the number of B-lines, with a pooled correlation coefficient of .08, .14, and .14, respectively. The correlation between lung ultrasound and dyspnea assessment (New York Heart Association class) was relatively high with a correlation coefficient of .57, but mainly due to the results in one article.^47^ This article had a Newcastle Ottawa quality assessment scale of 3/5. The correlation between B-lines and lung auscultation was moderate with a pooled correlation coefficient of .36. In addition, when the reduction in the number of B-lines and the weight change during dialysis were examined, correlation was moderate with a pooled *r* of .26 as shown in Table 2 and Figure 2. Heterogeneity was very low for all clinical parameters except for dyspnea assessment for which it was very high.

**Table 2.** Correlation of Lung Ultrasound Findings With Clinical and Paraclinical Measures.

Outcome	Pooled *r*	95% CI for *r*	*P* value
SBP-LUS	.08	0.01-0.16	.453
DBP-LUS	.14	0.04-0.24	.230
NYHA-LUS	.65	0.08-1.22	< .001
Delta weight-LUS	.27	0.07-0.47	.391
Edema-LUS	.14	0.03-0.32	.619
Lung auscultation-LUS	.38	0.21-0.56	.747
E/é-LUS	.28	0.04-0.53	.044
LVEF-LUS	.36	0.12-0.60	< .001
PulmP-LUS	.19	0.01-0.40	.175
LVMI-LUS	.19	0.06-0.33	.562
IVC Index-LUS	.32	0.15-0.48	.075
RVSP-LUS	.23	0.05-0.50	.1
BIA-LUS	.24	0.13-0.35	.447
BNP/pro-BNP-LUS	.50	0.33-0.67	.149

*Note.* CI = confidence interval; SBP = systolic blood pressure; LUS = lung ultrasound; DBP = diastolic blood pressure; NYHA = New York Heart Association; E/é = early filling to early diastolic mitral annular velocity; LVEF = left ventricular ejection fraction; PulmP = pulmonary pressure; LVMI = left ventricular mass index; IVC = inferior vena cava; RVSP = right ventricular systolic pressure; BIA = bioelectrical impedance analysis; BNP = brain natriuretic peptide.

### Correlation Between Lung Ultrasound and Echocardiographic Measurements

**Figure 3.**
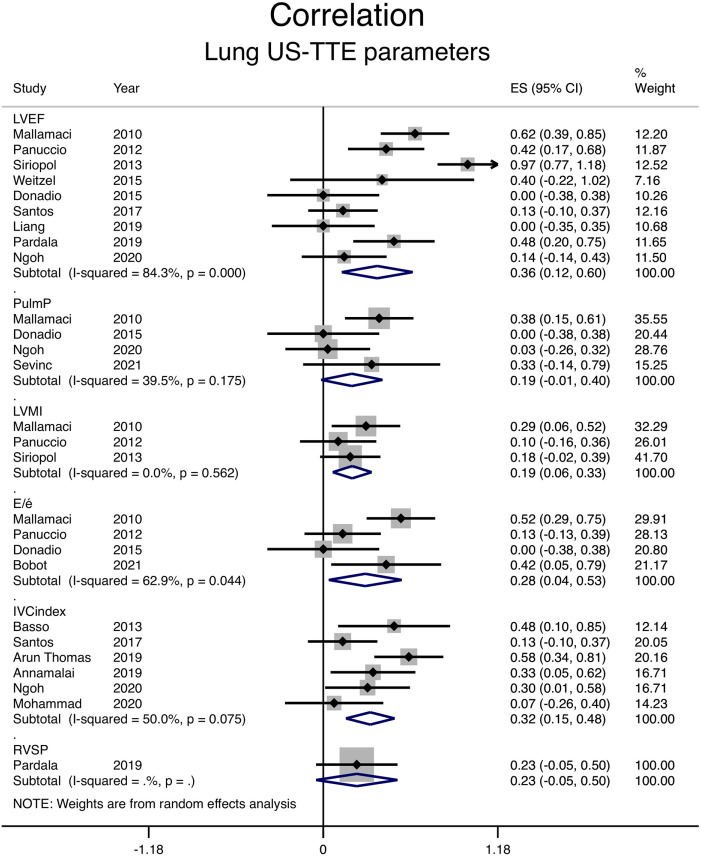
Correlation between the number of B-lines on lung ultrasound and different echocardiographic measurements. *Note.* CI = confidence interval; LVEF = left ventricular ejection fraction; PulmP = pulmonary pressure; LVMI = left ventricular mass index; E/é = early filling to early diastolic mitral annular velocity; IVC = inferior vena cava; RVSP = right ventricular systolic pressure; ES = Effect size.

Among echocardiographic parameters, LVEF and IVC index had the strongest correlation with the number of B-lines, with a pooled *r* coefficient of 0.35 and 0.31, respectively (shown in Figure 3 and Table 2). Correlation with diastolic dysfunction, as assessed by the E/é ratio, was weaker with a pooled *r* coefficient of 0.27. For LVMI and pulmonary artery pressure, both correlation coefficients were 0.19 (shown in Figure 3 and Table 2). Right ventricular systolic pressure correlation with B-lines was reported by a single study with a coefficient of .23.^36^

### Correlation Between Lung Ultrasound and Paraclinical Examinations

**Figure 4.**
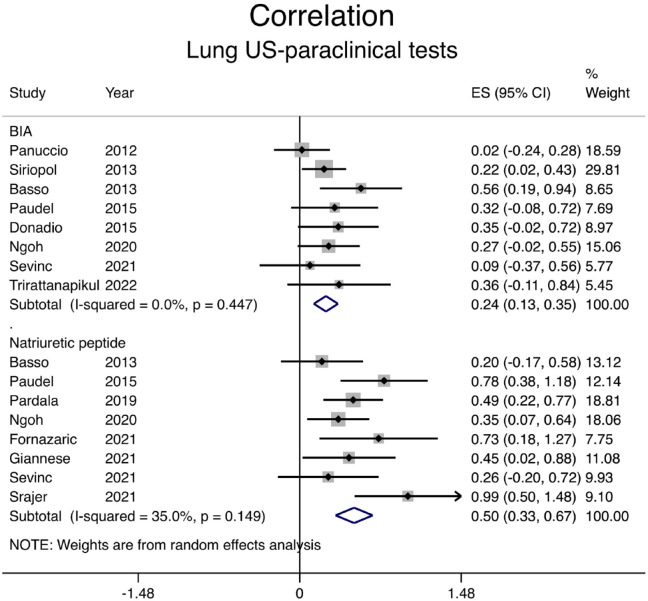
Correlation between the number of B-lines on lung ultrasound and paraclinical tests. *Note.* CI = confidence interval; BIA = bioelectrical impedance analysis; ES = Effect size.

Paraclinical examinations, such as bioimpedance techniques or natriuretic peptide levels, are commonly used to estimate volume status in patients with CKD. Eight studies compared bioimpedance techniques with lung ultrasound: correlation was weakly positive with a pooled correlation coefficient of .24 (shown in Figure 4 and Table 2). The correlation between natriuretic peptide levels (brain natriuretic peptide [BNP] or N-terminal pro-BNP) and sum of B-lines on ultrasound was stronger with a pooled correlation coefficient of .46 (shown in Figure 4 and Table 2).

### Clinical Outcomes (Observational Studies)

Data on clinical outcomes could not be pooled due to the very small number of events and/or the different methodologies the studies used in assessing volume status by lung ultrasound.

In a multicenter prospective study, Zoccali et al^4^ classified 392 patients on maintenance hemodialysis into 3 groups using the number of B-lines on lung ultrasound.

Patients with >60 B-lines on ultrasound had higher incidence of all-cause mortality and fatal or nonfatal cardiac events, compared with patients with <15 B-lines on ultrasound.

Siriopol et al^40^ prospectively followed 92 patients on maintenance hemodialysis for a median of 406 days. Patients were classified into 3 groups (<16, 16-30, or >30 lung comets) using lung ultrasound prior to the initiation of dialysis. In an adjusted Cox regression model, the hazard ratio for death from any cause was higher in patients with severe lung congestion (>30 comets on ultrasound) compared with the other 2 groups.

Saad et al prospectively followed 81 patients on maintenance dialysis who had been stratified into 3 groups using lung ultrasound (mild, moderate, or severe volume overload). Patients in the moderate or severe volume overload group had a higher incidence of death from any cause or major adverse cardiovascular events.^38^

In a prospective study by Beaubien-Souligny et al, 47 patients who were on hemodialysis for at least 3 months underwent lung ultrasound before and after 2 separate dialysis sessions to assess extravascular lung water. The authors used a simplified score (relative B-line score) to assess hydration status.^29^ Patients on the highest quartile of the postdialysis relative B-line score had a higher incidence of hospitalization for acute pulmonary edema or acute coronary syndrome, compared with patients on the second or third quartile.

Finally, Kawachi et al^32^ studied the association between lung congestion and mortality in patients undergoing maintenance hemodialysis. One-year survival was higher in patients with less pulmonary congestion: 55.4% versus 89.8% in the group of patients with >5 B-lines and <5 B-lines, respectively.

### Randomized Studies

Siriopol et al compared the effect of combining lung ultrasound and bioimpedance monitoring for dry weight assessment with standard of care on a composite outcome of death from any cause or cardiovascular events (including cardiovascular death, myocardial infarction, or stroke) in patients on maintenance hemodialysis with low cardiovascular risk. The study enrolled 250 participants.^18^ There was no significant difference between the 2 treatment strategies in this study.

Loutradis et al^50^ compared lung ultrasound with standard of care for adjusting the dry weight in a randomized study including 71 patients on maintenance hemodialysis who were hypertensive and considered to be euvolemic. Ultrafiltration was intensified in a higher percentage of patients who had lung ultrasound (54%), compared with patients in the usual care group (14%). The lung ultrasound-guided strategy was associated with decreased left and right atrial surfaces and with a decreased left ventricular E/é ratio, compared with the control arm. However, there was no difference between the 2 groups in left ventricular end diastolic volume or mass index. The lung ultrasound-guided strategy was also associated with better ambulatory BP control.^51^

The LUST trial enrolled 363 patients on maintenance hemodialysis with a high cardiovascular risk profile, as defined by history of myocardial infarction or heart failure.^19^ They were randomized to standard of care or a lung ultrasound-guided strategy. Lung ultrasound was performed by nephrologists before and after hemodialysis. The primary outcome, a composite of death, myocardial infarction, or heart failure, occurred in 34% of patients in the lung ultrasound group and 39% of patients in the control arm. A higher percentage of patient in the lung ultrasound arm achieved decongestion, defined as <15 B-lines.

## Discussion

This is the first systematic review, to our knowledge, comparing lung ultrasound with clinical, echocardiographic, and paraclinical assessment in patients on maintenance dialysis.

We identified a weak correlation between clinical, echocardiographic, or paraclinical examination findings and lung ultrasound findings in patients on maintenance dialysis. The only meaningful correlation was between change in number of B-lines or volume overload as detected by lung ultrasound and LVEF. We believe that the weak correlation identified between clinical or echocardiographic parameters and lung ultrasound is mostly due to important limitations of these techniques in assessing volume status, with lung ultrasound having higher accuracy in this population. Observational studies with lung ultrasound showed that this technique can identify patients with subclinical volume overload, and this might have prognostic implications, as patients with volume overload have worse clinical outcomes in this group of patients.^4,26-30,33-37,40,45,47-49^ The risk of bias was moderate for most observational studies. However, because the number of B-lines is an objective measure, we do not think that it could have introduced a serious risk of bias even if the outcome assessor was also aware of the intervention.

Three randomized studies compared a lung ultrasound-guided approach with standard of care on hard clinical endpoints in this population. Although patients in the lung ultrasound group achieved better decongestion or BP control, there was no difference between the 2 management strategies with respect to death from any cause or major adverse cardiovascular events. It is likely that causes of death might be much more complex in this population and that a single intervention, such as optimization of volume status, might not be sufficient to significantly affect hard outcomes, such as mortality or cardiovascular events. Whether this intervention may be associated with improved quality of life or exercise tolerance due to better decongestion has not been studied.

In addition, randomized studies might have been underpowered to detect a difference in hard clinical endpoints between the 2 studied arms. The study by Siriopol et al^18^ was powered to detect a difference in pulse wave velocity of 2 m/s, but this was not the primary outcome of the trial. The LUST trial had to be stopped early due to slow recruitment and enrolled only 77% of the 500 participants that were required to detect a significant difference in all-cause mortality, nonfatal myocardial infarction, or decompensated heart failure between the 2 study arms.^19^

There were no studies with lung ultrasound for volume assessment and management in patients with advanced CKD. Whether better volume control with lung ultrasound will be of any clinical benefit in this population remains to be established. In addition to cardiovascular outcomes and mortality, the effect of volume status management with lung ultrasound on CKD progression merits to be studied.

There are several limitations of our analysis. Heterogeneity was high for most echocardiographic parameters. Observational studies reporting clinical outcomes could not be pooled due to the very small number of events and/or the different methodologies they used in assessing volume status by lung ultrasound. In addition, lung ultrasound has not been standardized in this population: the number of measurements, B-line cutoffs, and scanning technique was highly variable across the included studies. The quality of the trials was variable. Furthermore, we only included studied published in PubMed and did not systematically review the gray literature in this topic.

In conclusion, lung ultrasound is a simple and noninvasive method that may be considered for the identification of patients with volume overload and may help for BP management. However, better volume control with lung ultrasound does not seem to be associated with improved hard clinical endpoints in this population.

## Supplemental Material

sj-docx-1-cjk-10.1177_20543581231217853 – Supplemental material for Volume Status Assessment by Lung Ultrasound in End-Stage Kidney Disease: A Systematic Review Click here for additional data file. Supplemental material, sj-docx-1-cjk-10.1177_20543581231217853 for Volume Status Assessment by Lung Ultrasound in End-Stage Kidney Disease: A Systematic Review by Aileen Kharat, Faissal Tallaa, Marc-Antoine Lepage, Emilie Trinh, Rita S. Suri and Thomas A. Mavrakanas in Canadian Journal of Kidney Health and Disease

sj-docx-2-cjk-10.1177_20543581231217853 – Supplemental material for Volume Status Assessment by Lung Ultrasound in End-Stage Kidney Disease: A Systematic Review Click here for additional data file. Supplemental material, sj-docx-2-cjk-10.1177_20543581231217853 for Volume Status Assessment by Lung Ultrasound in End-Stage Kidney Disease: A Systematic Review by Aileen Kharat, Faissal Tallaa, Marc-Antoine Lepage, Emilie Trinh, Rita S. Suri and Thomas A. Mavrakanas in Canadian Journal of Kidney Health and Disease

sj-docx-3-cjk-10.1177_20543581231217853 – Supplemental material for Volume Status Assessment by Lung Ultrasound in End-Stage Kidney Disease: A Systematic Review Click here for additional data file. Supplemental material, sj-docx-3-cjk-10.1177_20543581231217853 for Volume Status Assessment by Lung Ultrasound in End-Stage Kidney Disease: A Systematic Review by Aileen Kharat, Faissal Tallaa, Marc-Antoine Lepage, Emilie Trinh, Rita S. Suri and Thomas A. Mavrakanas in Canadian Journal of Kidney Health and Disease

sj-docx-4-cjk-10.1177_20543581231217853 – Supplemental material for Volume Status Assessment by Lung Ultrasound in End-Stage Kidney Disease: A Systematic Review Click here for additional data file. Supplemental material, sj-docx-4-cjk-10.1177_20543581231217853 for Volume Status Assessment by Lung Ultrasound in End-Stage Kidney Disease: A Systematic Review by Aileen Kharat, Faissal Tallaa, Marc-Antoine Lepage, Emilie Trinh, Rita S. Suri and Thomas A. Mavrakanas in Canadian Journal of Kidney Health and Disease
