# Supplementary material for: Volume Status Assessment by Lung Ultrasound in End-Stage Kidney Disease: A Systematic Review
Source: Can J Kidney Health Dis. 2023 Dec 25;10:20543581231217853. doi: 10.1177/20543581231217853 (PMC10750529; doi:10.1177/20543581231217853)
Supplement: sj-docx-1-cjk-10.1177_20543581231217853 – Supplemental material for Volume Status Assessment by Lung Ultrasound in End-Stage Kidney Disease: A Systematic Review [file sj-docx-1-cjk-10.1177_20543581231217853.docx]

**Supplementary file**

**Supplementary file 1.** PRISMA Checklist

**Supplementary file 2.** Risk of bias for randomized controlled trials

**Supplementary file 3.** Risk of bias for observational studies
