# Supplementary figures and images for: Volume Status Assessment by Lung Ultrasound in End-Stage Kidney Disease: A Systematic Review
Source: Can J Kidney Health Dis. 2023 Dec 25;10:20543581231217853. doi: 10.1177/20543581231217853 (PMC10750529; doi:10.1177/20543581231217853)

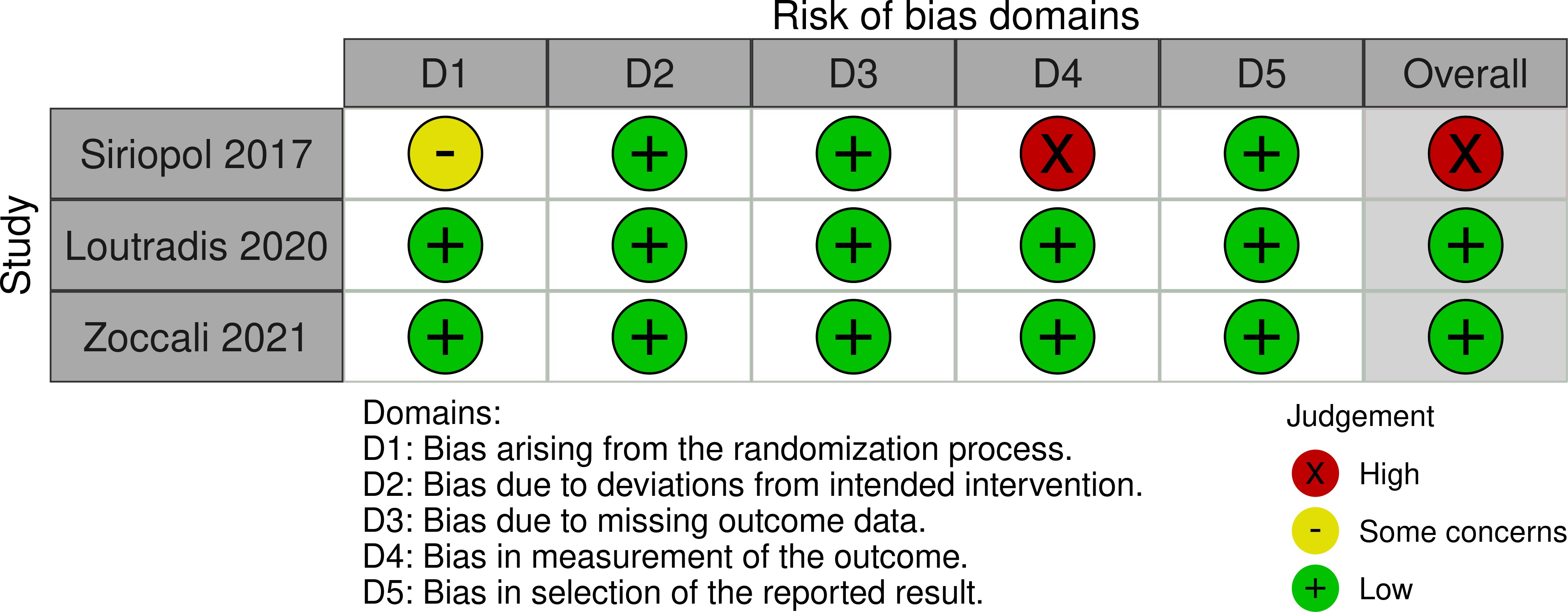

Supplement: sj-docx-3-cjk-10.1177_20543581231217853 – Supplemental material for Volume Status Assessment by Lung Ultrasound in End-Stage Kidney Disease: A Systematic Review [file sj-docx-3-cjk-10.1177_20543581231217853.docx]

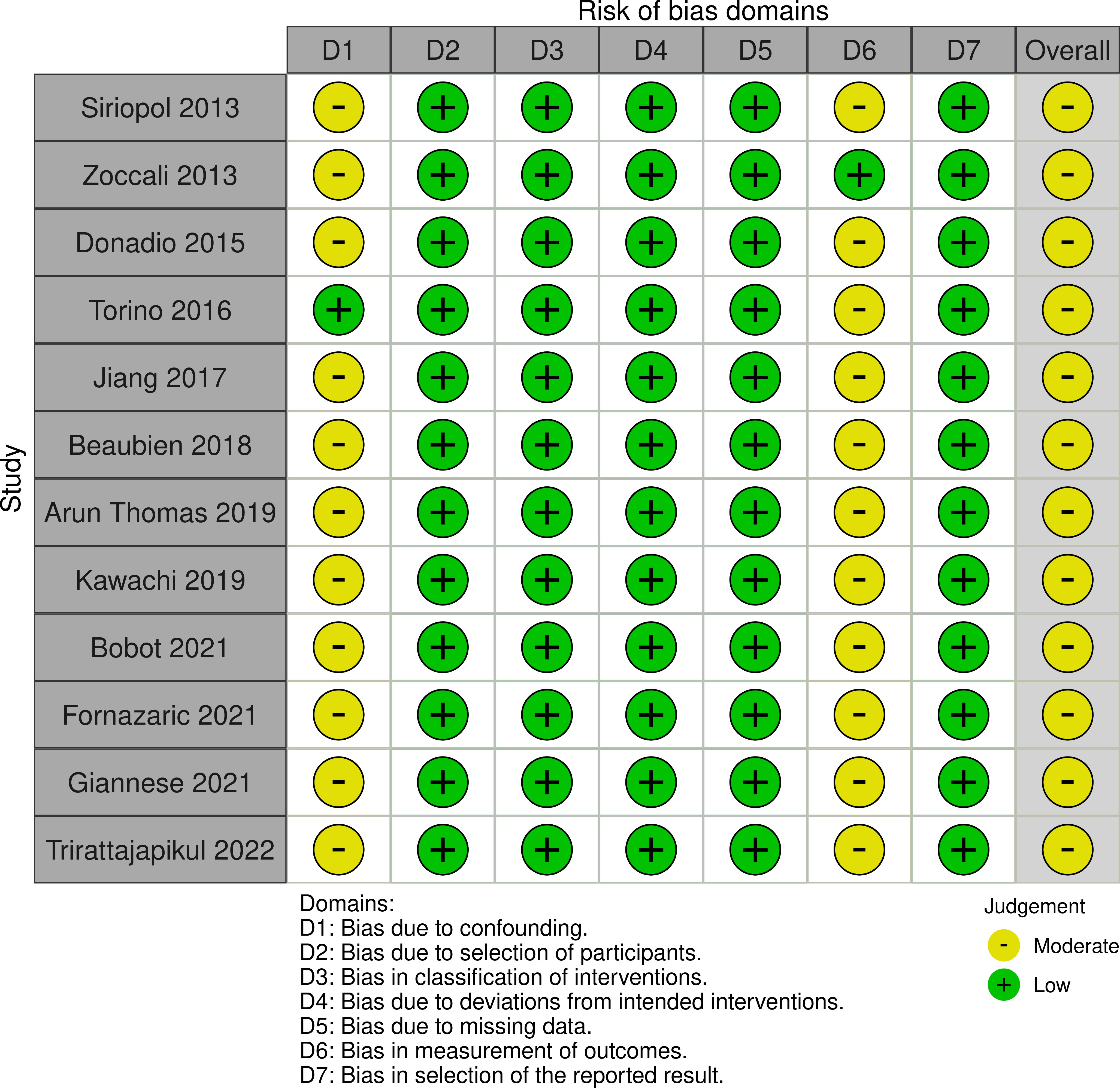

Supplement: sj-docx-4-cjk-10.1177_20543581231217853 – Supplemental material for Volume Status Assessment by Lung Ultrasound in End-Stage Kidney Disease: A Systematic Review [file sj-docx-4-cjk-10.1177_20543581231217853.docx]
